# Neuronal connectivity, behavioral, and transcriptional alterations associated with the loss of MARK2

**DOI:** 10.1096/fj.202400454R

**Published:** 2024-10-31

**Authors:** Hanna O. Caiola, Qian Wu, Junlong Li, Xue-Feng Wang, Shaili Soni, Kevin Monahan, George C. Wagner, Zhiping P. Pang, Huaye Zhang

**Affiliations:** 1 Department of Neuroscience and Cell Biology, Rutgers Robert Wood Johnson Medical School, Piscataway, New Jersey, USA; 2 Child Health Institute of New Jersey, New Brunswick, New Jersey, USA; 3 Department of Molecular Biology and Biochemistry, Rutgers University, Piscataway, New Jersey, USA; 4 Department of Psychology, Rutgers University, Piscataway, New Jersey, USA

**Keywords:** autism spectrum disorder, dendritic spines, epilepsy, hyperexcitability, learning and memory, MARK2, Par1, RNAseq, synapses

## Abstract

Neuronal connectivity is essential for adaptive brain responses and can be modulated by dendritic spine plasticity and the intrinsic excitability of individual neurons. Dysregulation of these processes can lead to aberrant neuronal activity, which has been associated with numerous neurological disorders including autism, epilepsy, and Alzheimer's disease. Nonetheless, the molecular mechanisms underlying abnormal neuronal connectivity remain unclear. We previously found that the serine/threonine kinase Microtubule Affinity Regulating Kinase 2 (MARK2), also known as Partitioning Defective 1b (Par1b), is important for the formation of dendritic spines in vitro. However, despite its genetic association with several neurological disorders, the in vivo impact of MARK2 on neuronal connectivity and cognitive functions remains unclear. Here, we demonstrate that the loss of MARK2 in vivo results in changes to dendritic spine morphology, which in turn leads to a decrease in excitatory synaptic transmission. Additionally, the loss of MARK2 produces substantial impairments in learning and memory, reduced anxiety, and defective social behavior. Notably, MARK2 deficiency results in heightened seizure susceptibility. Consistent with this observation, electrophysiological analysis of hippocampal slices indicates underlying neuronal hyperexcitability in MARK2-deficient neurons. Finally, RNAseq analysis reveals transcriptional changes in genes regulating synaptic transmission and ion homeostasis. These results underscore the in vivo role of MARK2 in governing synaptic connectivity, neuronal excitability, and cognitive functions.

## 1 | INTRODUCTION

The precise establishment and refinement of neuronal connectivity are critical for the brain to adapt and respond to its environment. In the human brain, neurons communicate through trillions of synaptic connections, leading to the formation of complex neuronal ensembles that mediate higher order cognitive functions such as learning and memory. Most excitatory synapses are formed on dendritic spines, which are small protrusions on dendrites that play key roles in modulating connectivity in the brain.^1^ Neural network connectivity is also intricately regulated by the intrinsic excitability of neurons. Increased excitability can facilitate the activation of subthreshold synaptic connections, which in turn may strengthen the connection and support memory formation.^2^ However, maladaptive changes in neuronal connectivity and excitability can lead to neurological disorders such as autism spectrum disorders (ASD),^3,4^ epilepsy,^5^ and Alzheimer’s disease (AD).^6–8^ Nonetheless, the molecular mechanisms underlying neuronal connectivity and how dysregulation of this process may lead to neurological disorders remain unclear.

We previously found that Microtubule Affinity Regulating Kinase 2 (MARK2), also known as Partitioning Defective 1b (Par1b), is important for the formation of dendritic spines in primary hippocampal neurons.^9^ MARK2 is a serine/threonine kinase that has key roles in cell polarity establishment and microtubule dynamics in many different cellular contexts.^10–14^ MARK2 is expressed at high levels in the brain and has been implicated in several neurological disorders. In particular, *de novo* loss-of-function mutations in *MARK2* have been identified in several genome-wide studies of ASD risk genes.^15–17^ *MARK2 de novo* mutations are also associated with bipolar disorder,^18^ schizophrenia,^19^ major depressive disorders,^20^ and epilepsy.^21^ Furthermore, MARK2 phosphorylation of tau is believed to play a role in initiating the tau hyperphosphorylation cascade in AD, and *MARK2* was found to be associated with AD in genome wide association studies.^18,22,23^ Previous in vitro studies show MARK2 plays a role in establishing neuronal polarity and regulating dendritic spine formation through modulating microtubule dynamics and the synaptic scaffold.^9,23–27^ Moreover, MARK2 activity is required for proper neuronal migration.^28^ Nevertheless, while MARK2 loss-of-function has been strongly associated with a variety of neurological disorders, how the loss of MARK2 affects neuronal connectivity and cognitive functions in vivo has yet to be studied.

Here, we show that the loss of MARK2 in mice results in increased thin dendritic spines at the expense of mushroom spines, with a concomitant reduction in excitatory transmission in the hippocampus. Moreover, the loss of MARK2 produces profound behavioral defects including impaired learning and memory, reduced anxiety-like behaviors, and impaired social behavior. Surprisingly, we also found that the loss of MARK2 results in increased seizure susceptibility. Further electrophysiological analysis revealed this is likely due to increased intrinsic neuronal excitability. To understand molecular pathways that may contribute to these phenotypes, we performed RNAseq in MARK2 knockout (MARK2−/−) hippocampi and found that pathways involved with ion homeostasis and synaptic transmission are primarily dysregulated. Together, our results establish the in vivo roles of MARK2 in regulating synaptic connectivity and behavior and provide insight into the molecular mechanisms underlying the function of MARK2 in the brain.

## 2 | MATERIALS AND METHODS

### 2.1 | Animals

All animal experiments performed in this study were carried out in accordance with Rutgers-RWJMS Institutional Animal Care and Use Committee protocols. B6.129X1-*Mark2*^tm1Hpw^/J (stock# 009365)^29^ and B6.Cg-Tg (Thy1-YFP) HJrs/J (stock# 003782)^30^ mice were purchased from Jackson Laboratories. Mice were housed with free access to food and water in a temperature and humidity regulated room with a 12 hr. light/dark cycle.

The genotypes of the Par1b/MARK2 mice were determined with PCR using tail or ear genomic DNA with the following oligonucleotides: primer 1 MARK2-Common-F (5′-AGCCACCTCTGCTGACGAGCAGCC-3′), primer 2 MARK2-WT-R (5′-GCAGACTGAACCAGGACTTGGTGC −3′), and primer 3 MARK2-Mut-R (5′-ATGATCTGGACG AAGAGCATCAGG-3′). Primers 1 and 2 amplify a 310 bp fragment specific to the wild-type allele. Primers 1 and 3 amplify a 437 bp fragment specific to the mutant allele.

The genotypes of the Thy1-YFP mice were determined with PCR using the following oligonucleotides: primer 1 Transgene-R (5′-CGGTGGTGCAGATGAACTT-3′), primer 2 Transgene-F (5′-ACAGACACACACCCAGGACA-3′), primer 3 Internal Positive Control-F (5′-CTAGGCCAC AGAATTGAAAGATCT-3′), primer 4 Internal Positive Control-R (5′-GTAGGTGGAAATTCTAGCATCATCC-3′ ). Primers 1 and 2 amplify a 415 bp fragment specific to the transgene. Primers 3 and 4 amplify a 324 bp fragment specific to the internal positive control.

### 2.2 | Behavior

#### 2.2.1 | Locomotor activity

A standard plastic cage without bedding was placed in the Opto-Varimex Minor locomotor activity monitor. Locomotor behavior was recorded by the monitor based upon interruptions in horizontal and ambulatory photocell beam located throughout the cage. MARK2+/+ (*n* = 14), MARK2+/− (*n* = 12) and MARK2−/− (*n* = 10) mice were individually housed for 1 week prior to the 30-min test session.

#### 2.2.2 | Rotarod test

The rotarod test evaluates motor coordination and balance. A shortened latency for the mouse to fall from the rod indicates a deficit in motor coordination and balance. MARK2+/+ (*n* = 14), MARK2+/− (*n* = 12) and MARK2−/− (*n* = 10) mice were placed on the rotarod with a diameter of 6 cm, rotating at 12 revolutions per minute. The rotarod was 60 cm above a padded receptacle. The latency to fall from the rotarod was recorded for each mouse for three trials, with each trial lasting 60 s.

#### 2.2.3 | Elevated plus maze

The elevated plus maze (EPM) was used to measure anxiety-like behavior. More time spent in the open arms indicates decreased anxiety-like behavior. The EPM was placed 60 cm above the floor with two long closed arms (65 cm long and 8 cm wide), two short open arms (30 cm long and 9 cm wide), and a central neutral 5 cm by 5 cm square. Each mouse was placed in the center square and observed for 10 min. The number and times the animal crossed into an open arm were recorded. MARK2+/+ (*n* = 13), MARK2+/− (*n* = 12) and MARK2−/− (*n* = 8) mice were tested.

#### 2.2.4 | Morris water maze

The apparatus for the Morris water maze (MWM) consisted of a circular, steel tub (diameter: 110 cm; height: 59 cm), and for the purposes of behavioral scoring, was demarcated into four “imaginary” quadrants (quadrant 1: Q1; quadrant 2: Q2; quadrant 3: Q3; quadrant 4: Q4). The tub was filled with regular tap water and water temperature was maintained at approximately 21 ± 1°C. The water was made opaque by adding black, nontoxic, powder tempera paint (Rich Art, Northvale, NJ). The platform consisted of a small, clear, perforated, plexiglass disc (diameter: 9 cm) mounted onto a steel rod and affixed to a heavy metal base (height: 48 cm). MARK2+/+ (*n* = 12), MARK2+/− (*n* = 14) and MARK2−/− (*n* = 9) mice were tested for seven consecutive days, four trials per day. In each trial, a maximum swim time of 60 s was imposed. Each animal was subjected to 2 days of visible platform training followed by 4 days of a hidden platform test and a probe test on Day 7.

For the visible platform test (Day 1 and Day 2), the platform with a flag was raised 1 cm above the water. Animals were trained without the cues on the wall. The platform and start positions were randomly changed for each trial to avoid habituation to a particular quadrant.

For the hidden platform test (Day 3 through Day 6), the platform was submerged 1 cm below the surface of the water and placed in Q3. The start positions were randomly changed. Animals were trained with the cues on the wall. In each trial, a maximum swim time of 60 s was imposed. Between trials, a 10 s interval was imposed with the mouse on the platform. Spatial learning was measured as the time the mouse spent to find the platform. A shorter latency to find the platform represents better spatial learning.

For the probe test (Day 7), the platform was removed. During this probe trial the mouse was allowed to swim for 60 s. A significant increase in time spent in the target quadrant compared with all other quadrants suggests retention of spatial memory.

#### 2.2.5 | T-maze active avoidance test

Mice were tested in a T-maze consisting of two 20 × 11 cm chambers connected to a 40 × 10 cm corridor with 18 cm high walls made of plexiglass. The floor was made of stainless-steel bars spaced 0.75 cm apart and connected to a shock generator except in the “safe” chamber. In each trial, mice were placed in the start box with an initial interval of 20 s. A conditional stimulus tone accompanied by the opening of the start box door initiated the trial. Mice could avoid the shock by moving to the safe arm of the T-maze within 10 s of the tone. Failure to make an avoidance response led to onset of a 0.8 mA foot shock, which could be terminated by moving to the safe arm as an escape response (also 10 s maximum). Each animal underwent 10 trials per day, per mouse, per group (MARK2+/+ *n* = 10, MARK2+/− *n* = 10 and MARK2−/− *n* = 8). The type of response (avoidance or escape) and the latency for the animal to make either avoidance or escape responses were recorded.

#### 2.2.6 | Social chamber

The social chamber was a 40 cm × 40 cm × 36.6 cm plexiglass chamber with a stainless-steel grid floor. Within the chamber there were two cylinders 11 cm in diameter and 13 cm tall made of the same stainless-steel grid as the floor, located in opposite corners of the chamber. Each mouse (MARK2+/+ *n* = 9, MARK2−/− *n* = 6) was given a 10-min habituation period to explore the chamber before the start of the trial. After 10 min, one age- and sex-matched MARK2+/− mouse (target mouse) was placed in one of the cylinders and the subject mouse was placed in the center of the chamber. A contact was recorded each time the subject placed one or both paws on a cylinder. The number of contacts with either the target cylinder containing the target mouse or the empty control cylinder was recorded. More contact with the target cylinder containing a novel mouse compared to the empty cylinder indicated a higher level of social behavior.

#### 2.2.7 | Seizure susceptibility

MARK2+/+ (*n* = 13) and MARK2+/− (*n* = 14) mice were injected with pilocarpine (300 mg/kg) intraperitoneally to induce seizures. To reduce side effects from peripheral cholinergic inputs, mice were also injected with methyl scopolamine (1 mg/kg) 30 min prior to pilocarpine injection. Mice were recorded for 2 h following the injection and seizure behavior was scored by a blinded observer using the Racine scale as modified by Borges et al.^31,32^ Classifications were as follows: normal activity (stage 0); rigid posture or immobility (stage 1); stiffened, extended, and often arched tail (stage 2); partial body clonus, including forelimb or hind limb clonus or head bobbing (stage 3); whole body continuous clonic seizures with rearing (stage 4); severe whole body continuous clonic seizures with rearing and falling (stage 5); and tonic–clonic seizures with loss of posture or jumping (stage 6).

### 2.3 | Western blot

For Western blots of whole cell lysates, mice were euthanized via cervical dislocation and brains were dissected in ice cold 1× phosphate-buffered saline (PBS) prior to being flash frozen on dry ice or in liquid nitrogen. Whole brain tissue was lysed on ice in RIPA buffer containing 20 mM Tris-HCl (pH 7.4), 150 mM NaCl, 2mM EDTA, 2mM EGTA, 0.5% Nonidet P-40, 1% Triton X-100, 0.25% sodium deoxycholate and 10 mM DTT and supplemented with protease inhibitor cocktail (1:1000, Sigma Aldrich P-8340), phosphatase inhibitor cocktail (1:100, Sigma Aldrich P0044), 10 mM β-glycerophosphate, and 10 mM NaF. Lysates were cleared by centrifugation at 13 000*g* for 10 min at 4°C. Protein concentrations were measured with Pierce 600 nm Protein Assay Reagent (Thermo Scientific, 22660). Primary and secondary antibodies used are listed in Table S1. Proteins were visualized by enhanced chemiluminescence and imaged using a Syngene G:BOX iChemi XR system (Syngene USA, Frederick, MD) or Azure 600 imaging system (Azure Biosystems Inc., Dublin, CA). The expression was quantified in FIJI ImageJ using densitometry and normalized to GAPDH as a loading control.

### 2.4 | Synaptosomal fractionation

Synaptosomal fractions were prepared as described previously.^33,34^ Briefly, brains were dissected in ice-cold 1× PBS and flash frozen in liquid nitrogen. Tissue was homogenized with a 2 mL glass douncer in Buffer 1 (10 mM HEPES (pH 7.4), 2 mM EDTA, protease inhibitor cocktail (1:1000, Sigma Aldrich P-8340), phosphatase inhibitor cocktail (1:100, Sigma Aldrich P0044)). Following a 10-min incubation on ice, lysates were centrifuged for 10 min at 1000*g* at 4°C to create the P1 nuclei pellet and S1 homogenate supernatant. The S1 homogenate was then centrifuged for 15 min at 10 000*g* at 4°C to create S2 cytosolic supernatant and the P2 crude synaptosome pellet. P2 was resuspended in Buffer 2 (50 mM HEPES (pH 7.4), 2 mM EDTA, 2 mM EGTA, 1% Triton-X-100, protease inhibitor cocktail (1:1000, Sigma Aldrich P8340), phosphatase inhibitor cocktail (1:100, Sigma Aldrich P0044)). Protein concentrations were measured with Pierce 660 nm Protein Assay Reagent (Thermo Scientific, 22660) and prepared in Laemmli’s sample buffer. Primary and secondary antibodies used are listed in Table S1. Expression was quantified in FIJI ImageJ using densitometry and normalized to total protein using No-Stain Protein Labeling Reagent (Invitrogen, A44449).

### 2.5 | Dendritic spine analysis

MARK2/YFP-H mice were perfused with ice-cold PBS and 4% paraformaldehyde (PFA) followed by a post fixation in 4% PFA overnight in 4°C. Brains were subjected to increasing concentrations of sucrose solutions (10%, 20% and 30%), each concentration for 24 h in 4°C. The brains were then sectioned at 70 μm using a microtome. Following washes in 1× PBS, sections were mounted using Vectashield with DAPI (Vector Laboratories, Burlingame, CA).

Fluorescence images were acquired using an Olympus FV1000 confocal microscope with a 60X water-immersion lens (NA 1.00, Olympus, Center Valley, PA). Z-stacks of dendritic spines were taken in 0.5 μm steps at 60× from the *stratum radiatum* layer of the hippocampus. Images of dendritic spines were blinded to the experimenter analyzing the images, and the morphology and density were measured using Reconstruct.^35^ Spine length was defined as the length from the tip of the spine head to the point where the spine joins the dendrite. Spine width was defined as the maximum width of the spine head perpendicular to the long axis of the spine neck.

### 2.6 | Electrophysiology

Brain slice physiology was performed as described previously^36^ with modifications. Briefly, mice were deeply anesthetized with Euthasol. Brains were removed and quickly immersed in cold (4°C) oxygenated cutting solution containing the following (in mM): 50 sucrose, 2.5 KCl, 0.625 CaCl_2_, 1.2 MgCl_2_, 1.25 NaH_2_PO_4_, 25 NaHCO_3_, and 2.5 glucose (oxygenated with 95%O_2_/5%CO_2_). Coronal hippocampal slices, 300 μm in thickness, were cut using a vibratome (catalog #VT 1200S, Leica). Brain slices were collected in artificial CSF (ACSF) and bubbled with 5% CO_2_/95% O_2_. The ACSF contained the following (in mM): 125 NaCl, 2.5 KCl, 2.5 CaCl_2_, 1.2 MgCl_2_, 1.25 NaH_2_PO_4_, 26 NaHCO_3_, and 2.5 glucose, bubbled with 95%O_2_/5%CO_2_. After 60 min of recovery, slices were transferred to a recording chamber and perfused continuously at 2–4 mL/min with oxygenated ACSF at 30°C.

To record mini excitatory postsynaptic currents (mEP-SCs), picrotoxin (50 μM, Sigma Aldrich, Saint Louis, MO) was added to block inhibitory currents mediated by GABA receptors and tetrodotoxin (1 μM, Abcam Biochemicals) was added to block action potentials. Patch pipettes (3.8– 4.4 MΩ) were pulled from borosilicate glass (G150TF-4; Warner Instruments) and filled with internal solution containing (in mM): 40 CsCl, 10 HEPES, 0.05EGTA, 1.8 NaCl, 3.5 KCl, 1.7 MgCl_2_, 2 Mg ATP, 0.4 Na_4_GTP, 10 Phosphocreatine (pH 7.2, 280–290 mOsm). EPSCs were recorded in whole-cell voltage clamp mode (Axon 100B amplifier, Molecular Devices), filtered at 2 kHz, digitized at 10 kHz, and collected using Clampex 10.2 (Molecular Devices). Neurons were held at −70 mV.

To record intrinsic excitability of pyramidal neurons, slices were bathed in ACSF at 30°C. The 1 s currents were injected into neurons in 2 s intervals and 10 pA steps from −20 to 160 pA with 4–6 MΩ patch pipettes filled with internal solution (in mM: 126 K-Gluconate, 4 KCl, 10 HEPES, 4 MgATP, 0.3 Na2GTP, 10 Phosphocreatine, 263 mOsm, pH 7.2). Active and passive properties were measured in whole-cell current clamp mode (Multiclamp 700B amplifier, Molecular Devices), digitized at 20 kHz, and collected using Clampex 10.2 (Molecular Devices).

### 2.7 | RNA-seq

MARK2+/+ (*n* = 4) and MARK2−/− (*n* = 5) mice were sacrificed at 8 weeks old via live cervical dislocation followed by hippocampal dissection on ice-cold PBS and flash freezing in liquid nitrogen or dry ice. Hippocampal samples were stored at −80°C until extraction, library preparation, and sequencing (performed at Admera Health, South Plainfield, NJ). Libraries were prepared with NEBNext UltraII (non-directional) kit with Poly A selection. Data was processed using 150-bp paired end reads. Adaptor sequences were removed from raw sequences using CutAdapt^37^ and the remaining reads were aligned to the mm10 mouse genome (NCBI RefSeq Assembly GCF_000001635.20) using STAR v.2.7.10a.^38^ Reads with mapping quality below 30 were removed with Samtools.^39^ RNA-seq data analysis was performed with DESeq2^40^ in RStudio. Genes with low expression (less than 20 counts) were excluded. DESeq2 was also used to calculate TPM, FPKM, fold changes, *p* values, and adjusted *p* values (*p*_adj_). *p*_adj_ significance threshold was .05. Heatmaps (pheatmap),^41^ MA (ggplot2),^42,43^ and volcano (ggplot2) plots were created in RStudio. Gene ontology (GO) analysis was performed using DAVID.^44,45^ Protein–protein interaction networks were created with the STRING database using Cytoscape^46^ and MCL clustering with AutoAnnotate.^47^

### 2.8 | Experimental design and statistical analyses

All statistical analyses were performed using GraphPad Prism. All datasets were tested for normality. If not normal, ROUT (*Q* = 1%) was used to normalize. Nonparametric tests were used if no outliers were found. Unpaired *t*-tests or Mann–Whitney *U* tests were used to compare between two groups. Ordinary one-way ANOVA, Kruskal–Wallis, or Brown–Forsythe, were used for comparisons between three or more groups followed by Tukey’s *post-hoc* test. Nested one-way ANOVAs were used in place of ordinary one-way ANOVA for the spine analyses of length, width, length-to-width ratio (LWR), and spine density to account for individual mouse differences. Two-way ANOVA was used for spine morphology analysis followed by Tukey’s *post-hoc*. Repeated measures two-way ANOVA was used for behavioral tests that spanned multiple time points followed by Dunnett’s *post-hoc* test. Both male and female mice were used in all experiments unless otherwise specified.

## 3 | RESULTS

### 3.1 | Expression of MARK family proteins in MARK2 knockout mice

**FIGURE 1:**
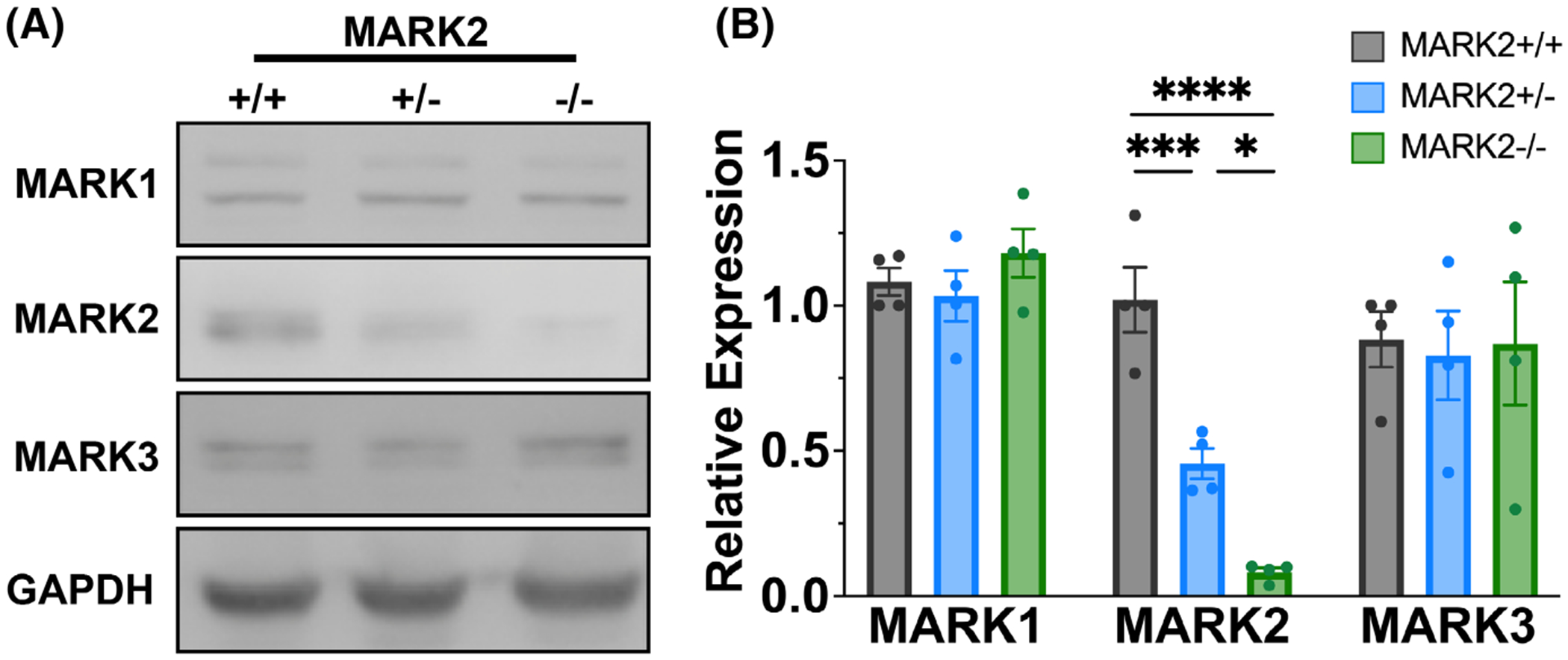
Expression of MARK family proteins in MARK2 knockout mice. (A) Representative blot of MARK1, MARK2, and MARK3 protein expression in MARK2+/+ (*n* = 4), MARK2+/− (*n* = 4), and MARK2−/− (*n* = 4) mice normalized to GAPDH. The faint residual band in the MARK2 blot in the MARK2−/− brains is likely due to non-specific interactions with other MARK family members. (B) Quantification of MARK protein expression from Western blot in (A). One-way ANOVA was performed followed by Tukey’s *post-hoc* test: MARK1 (*F*(2, 9) = 1.107, *p* = .3397), MARK2 (*F*(2, 9) = 43.39, *p* < .0001), and MARK3 (*F*(2, 9) = 0.03143, *p* = .9692). *p < .0332, ***p < .0002, ****p < .0001. Plot shows mean +/− SEM. Full statistics details can be found in Table S1–1.

To examine the in vivo functions of MARK2 in neuronal connectivity and behavior, we used *Mark2* knockout mice (Mark2−/−) (B6.129X1-*Mark2*^tm1Hpw^/J).^29^ In mammals, there are four Par1/MARK members.^13^ To determine whether knockout of Par1b/MARK2 in vivo causes compensatory expression of other Par1/MARK family members in the brain, we extracted total protein from forebrain tissue of 5- to 8-week-old MARK2 wildtype (+/+), heterozygote (+/−), and knockout mice and examined the expression level of different Par1/MARK family members via Western blot. Expression of Par1b/MARK2 was partially decreased in MARK2+/− mice and was abolished in MARK2−/− mice (Figure 1A,B, Table S1–1). The faint residual band in the MARK2 blot in the MARK2−/− brains is likely due to non-specific interactions with other MARK family members. Expression of Par1c/MARK1 or Par1a/MARK3 was not significantly altered in either the MARK2+/− or MARK2−/− mice (Figure 1). Par1d/MARK4 is expressed at very low levels in the brain^48^ and therefore could not be reliably detected with Western blot. Altogether, these results confirm that MARK2 is decreased as expected in MARK2+/− and MARK2−/− animals while other MARK family members are not affected.

### 3.2 | Loss of MARK2 leads to defects in dendritic spine morphogenesis and synaptic transmission in the hippocampus

Dendritic spines are sites of excitatory input in the nervous system. Changes to dendritic spine density, size, and shape have been linked to numerous neurological disorders.^49,50^ The size and shape of spines range from immature filipodia, which are long, thin spines to mature mushroom-shaped spines, which are shorter and wider.^51^ Furthermore, mushroom-shaped spines generally contain a larger postsynaptic density with an increase in AMPA receptors.^52,53^ Therefore, mushroom spines are considered more mature than longer and thinner spines due to their increased synaptic strength.^54^

**FIGURE 2:**
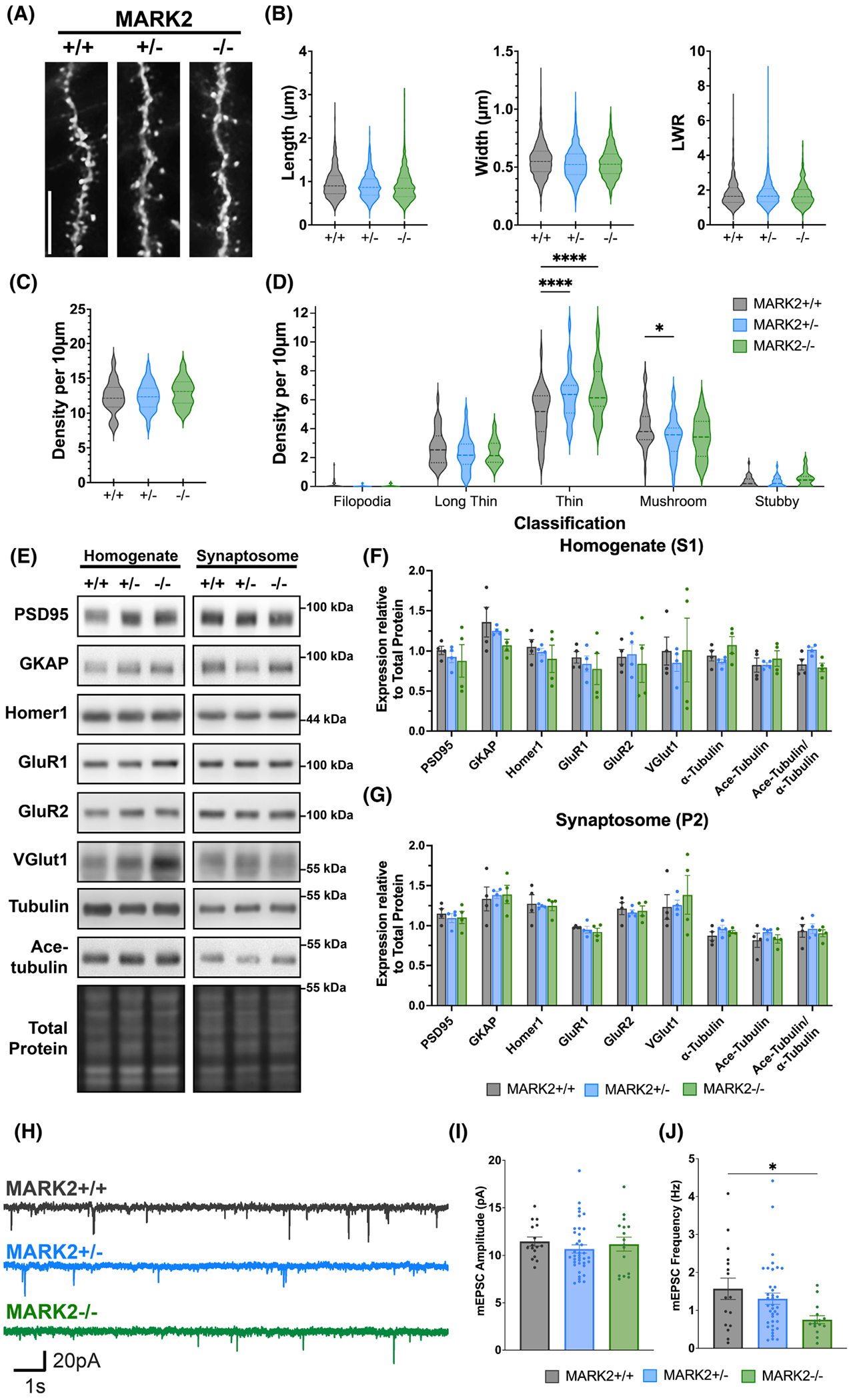
Loss of MARK2 leads to defects in dendritic spine morphogenesis and synaptic transmission in the hippocampus. (A) Representative images of secondary dendrites of pyramidal neurons in *strata radiatum* layer of the hippocampus from MARK2+/+ (*n* = 4 mice, 35 dendrites, 1858 spines), MARK2+/− (*n* = 4 mice, 31 dendrites, 1642 spines), and MARK2−/− (*n* = 2 mice, 16 dendrites, 911 spines) mice. Scale bar: 10 μm. (B) No changes in spine length (*F*(2, 7) = 2.242, *p* = .1768), spine head width (*F*(2, 7) = 2.038, *p* = .2006), and length-to-width ratio (LWR; *F*(2, 7) = 0.4097, *p* = .6789) were detected between genotypes by nested one-way ANOVA. (C) No changes in overall spine density were observed between genotypes by nested one-way ANOVA (*F*(2, 7) = 0.1386, *p* = .8729). (D) Spines were morphologically classified sequentially using the following criteria: Filipodia (length >2 μm), mushroom (head width >0.6 μm), long thin (length >1 μm), thin (LWR >1 μm), or stubby (LWR <1 μm). MARK2+/− and MARK2−/− dendrites had fewer mushroom spines and increased thin spines. Two-way ANOVA followed by Tukey’s post hoc. (E) Representative blots of synaptic scaffolding, excitatory transmission machinery, and cytoskeletal components in whole-cell homogenate and crude synaptosomes of MARK2+/+ (*n* = 4), MARK2+/− (*n* = 4), and MARK2−/− (*n* = 4) mice. (F, G) Quantification of protein expression in homogenate (F) or crude synaptosomes (G) across genotypes. Values are normalized to total protein. Ordinary one-way ANOVA or Brown–Forsythe ANOVA, as appropriate (see Table S1–1 for full statistics). (H) Representative mEPSC traces from hippocampal neurons of MARK2+/+, MARK2+/−, and MARK2−/− mice. (I) mEPSC amplitude compared between MARK2+/+ (*n* = 16), MARK2+/− (*n* = 39), and MARK2−/− (*n* = 16) neurons. Kruskal–Wallis ANOVA (stat = 5.242, *p* = .1549). (J) mEPSC frequency between MARK2+/+, MARK2+/−, and MARK2−/− neurons. Brown–Forsythe ANOVA (F*(2, 32.10) = 3.678, *p* = .0364) followed by Dunnet’s T3 post hoc test. *p < .0332, ****p < .0001. Mean +/− SEM. Full statistics details can be found in Table S1–1.

Previously, we and others have shown that Par1/MARK is necessary for spine morphogenesis in cultured hippocampal neurons.^9,27^ To explore whether MARK2 plays a role in spine morphogenesis in vivo, we crossed MARK2 mice with Thy1-YFP mice to create MARK2/YFP-H mice, in which layer V pyramidal neurons in the cortex and pyramidal neurons in the hippocampus are positive for YFP. Dendritic spines of dorsal CA1 pyramidal neurons on the secondary apical dendrites in the *strata radiatum* layer were measured for density, length, width, and morphological classification (Figure 2A, Table S1–1). We found no differences across genotypes for length, width (Figure 2B), or overall spine density (Figure 2C). Interestingly, we found that MARK2+/− and MARK2−/− mice have an increased number of thin spines and reduced number of mushroom spines (Figure 2D). These results suggest that while the overall density of dendritic spines is not altered, there is a shift towards immature spines with the loss of MARK2.

To understand if the observed changes in dendritic spine morphology could be explained by the underlying dysregulation of postsynaptic density components, we used synaptosomal fractionation to probe for scaffolding proteins, cytoskeletal components, and excitatory transmission machinery (Figure 2E). Surprisingly, we found no changes in total (Figure 2F) or synaptic levels (Figure 2G) of any of the targeted proteins across genotypes.

Since synaptosomal levels of receptors and scaffolding proteins may not fully reflect functional changes in synaptic transmission, we aimed to understand if there were any functional defects using electrophysiology. Therefore, we measured miniature excitatory postsynaptic currents (mEPSCs) in hippocampal pyramidal neurons via whole-cell voltage clamp in acute brain slices (Figure 2H). We found no changes in mEPSC amplitude (Figure 2I); however, we observed a significant decrease in mEPSC frequency in MARK2−/− mice when compared with MARK2+/+ mice (Figure 2J). Together, these results suggest that loss of MARK2 results in a net reduction in functional synapses as measured by mEPSC frequency. This is in line with the observed reduction of mushroom-shaped spines. Furthermore, the absence of any mEPSC amplitude changes is consistent with our findings that the synaptosomal levels of receptors and scaffolding proteins are largely unaltered.

### 3.3 | Loss of MARK2 results in deficits in hippocampal-dependent learning and memory tasks

**FIGURE 3:**
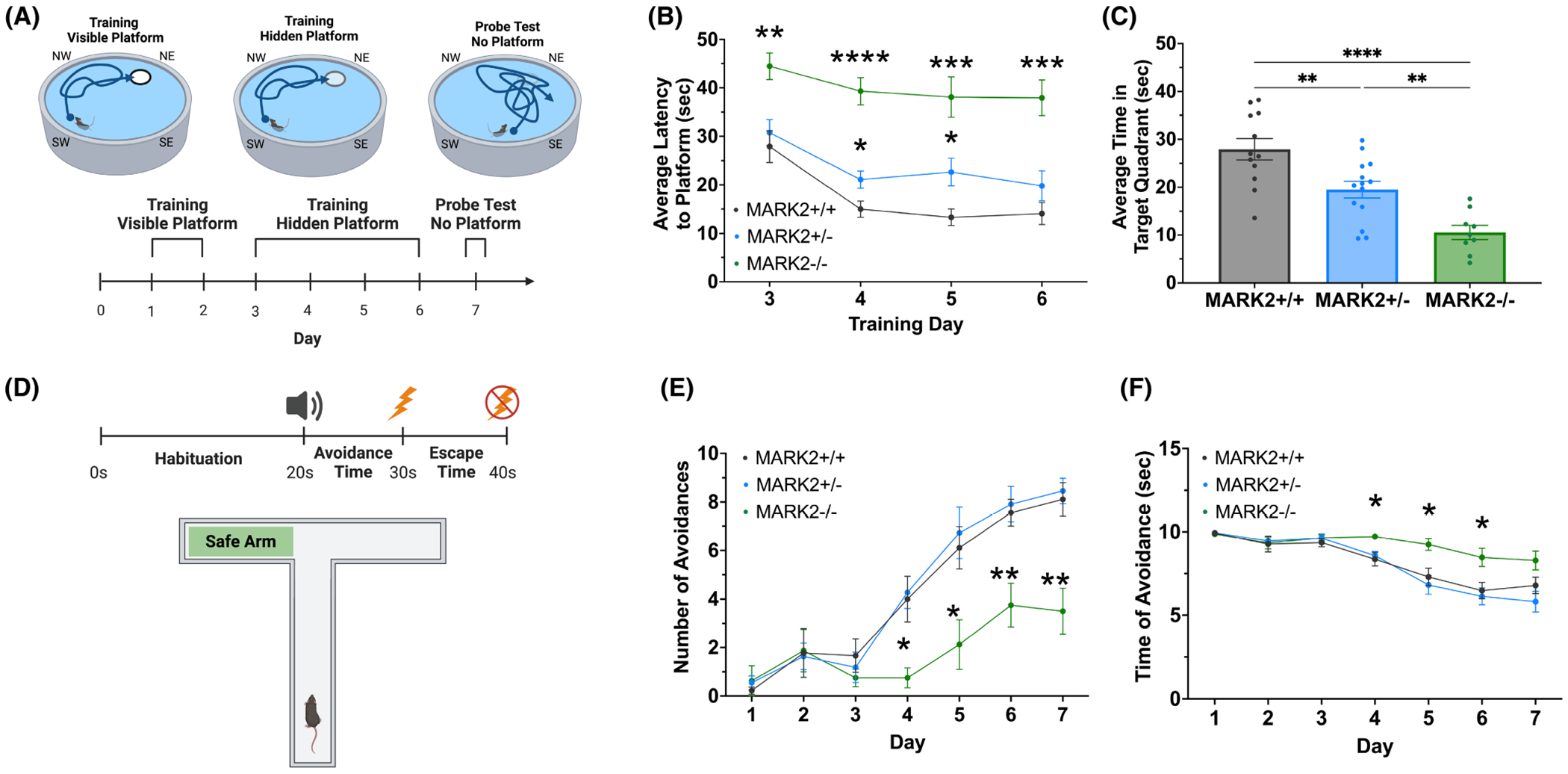
Loss of MARK2 results in deficits in hippocampal-dependent learning and memory tasks. (A) Experimental timeline of MWM training. The platform was visible during Days 1 and 2 of training and then hidden during Days 3 through 6. The platform was removed completely during the probe test. Each animal underwent 4 trials per day. (B) Average latency to platform in seconds on training days 3 through 6 (hidden platform) between MARK2+/+ (*n* = 12), MARK2+/− (*n* = 14), and MARK2−/− (*n* = 9) mice. Two-way repeated measures ANOVA (Genotype: *F*(2, 32) = 37.62, *p* < .0001) followed by Dunnet’s *post-hoc* test. (C) Performance of MARK2+/+ (*n* = 12), MARK2+/− (*n* = 14), and MARK2−/− (*n* = 9) mice in probe test on Day 7. Ordinary one-way ANOVA (*F*(2, 32) = 18.50, *p* < .0001) followed by Tukey’s *post-hoc*. (D) Experimental timeline of each active avoidance task trial. Mice were placed into a T-maze and allowed to habituate. After 20 s of habituation, a tone initiated the trial and the door to the safe arm opened. After 10 s, a 0.8 mA foot shock was administered for an additional 10 s. The mice were considered to have successfully avoided the shock if they reached the safe arm prior to shock onset. Each mouse underwent 10 trials per day. (E) Number of times MARK2+/+ (*n* = 10), MARK2+/− (*n* = 10), and MARK2−/− (*n* = 8) mice avoided the shock. Two-way repeated measures ANOVA (Genotype: *F*(2, 25) = 8.545, *p* = .0015) followed by Dunnet’s *post-hoc* test. (F) Time it took for MARK2+/+, MARK2+/−, and MARK2−/− mice to reach the safe arm after the tone was played. Two-way repeated measures ANOVA (Genotype: *F*(2, 25) = 5.859, *p* = .0082) followed by Dunnet’s *post-hoc* test. *p < .0332, **p < .0021, ***p < .0002, ****p < .0001. Mean +/− SEM. Full statistics details can be found in Table S1–1.

Changes to dendritic spines and synaptic transmission are often correlated with changes at the behavioral level. In particular, proper regulation of dendritic spines and synaptic transmission is critical in learning and memory tasks.^55,56^ Therefore, we aimed to understand whether MARK2+/− and MARK2−/− mice exhibit deficits in learning and memory. First, we used the hippocampal-dependent learning task MWM. MARK2+/+, MARK2+/−, and MARK2−/− mice were trained for 2 days with the platform visible and then for an additional 4 days with the platform hidden under the surface of the water with each training day consisting of 4 trials (Figure 3A). During training days 1 and 2 in which the platform was visible, all mice successfully found the platform. Importantly, MARK2−/− mice performed similarly to MARK2+/+ and MARK2+/− mice during the first three trials of Day 1 in which the platform was visible, indicating no motor or visual impairments that may impede their performance (Figure S1). Both MARK2+/+ and MARK2+/− mice showed improved performance by the last trial and a decreased latency to platform as compared with earlier trials, whereas MARK2−/− mice showed an increased latency to platform compared to MARK2+/+ and MARK2+/− mice (Figure S1), indicating a learning impairment in the MARK2−/− mice even in the visible trials. Once the platform was hidden on training Day 3 through Day 6, MARK2−/− mice consistently exhibited increased latency to platform compared to MARK2+/+ mice (Figure 3B, Table S1–1). MARK2+/− mice exhibited increased latency to platform compared to controls only on Days 4 and 5 (Figure 3B). During the probe test on Day 7 in which the platform was removed from the tank, MARK2−/− mice spent significantly less time in the target quadrant compared to the MARK2+/+ and MARK2+/− mice (Figure 3C). MARK2+/− mice also performed significantly worse in the probe test compared to MARK2+/+ mice (Figure 3C).

Next, we performed another hippocampal-dependent task, the T-maze active avoidance test (Figure 3D). The mice were subjected to 10 trials per day. Each trial consisted of an initial interval of 20 s after which a tone was played to initiate the trial. The mice had 10 s to reach the escape arm, which would avoid a 10 s 0.8 mA foot shock. If the mice failed to avoid the shock, they could escape after the shock onset by moving to the escape arm. Mice were considered to have learned the task if they successfully paired the conditioned stimulus (tone) with the unconditioned stimulus (shock) and exhibited avoidance behavior.

Given the impaired performance of the MARK2−/− mice in the MWM, we hypothesized that these mice would have increased avoidance times and reduced number of avoidances compared to MARK2+/+ and MARK2+/− mice. We found that MARK2+/+ and MARK2+/− mice successfully learned to associate the tone and shock by Day 4 as evidenced by an increased number of avoidances (Figure 3E) and decreased avoidance times (Figure 3F), which continued to trend in the same respective directions through Day 7. Interestingly, in the MARK2−/− mice, the total number of avoidances was significantly lower from Day 4 through Day 7 compared to MARK2+/+ and MARK2+/− (Figure 3E). Additionally, it took the MARK2−/− mice significantly longer to avoid the shock (Figure 3F). To ensure that the poor performance of the MARK2−/− mice was not a result of decreased motor or locomotor activity, the mice were also subjected to rotarod and locomotor tests in which we found no differences between genotypes (Figure S2). In sum, these experiments indicate that MARK2 is necessary for proper learning and memory formation.

### 3.4 | MARK2−/− mice show reduced sociability and reduced anxiety-like behavior

**FIGURE 4:**
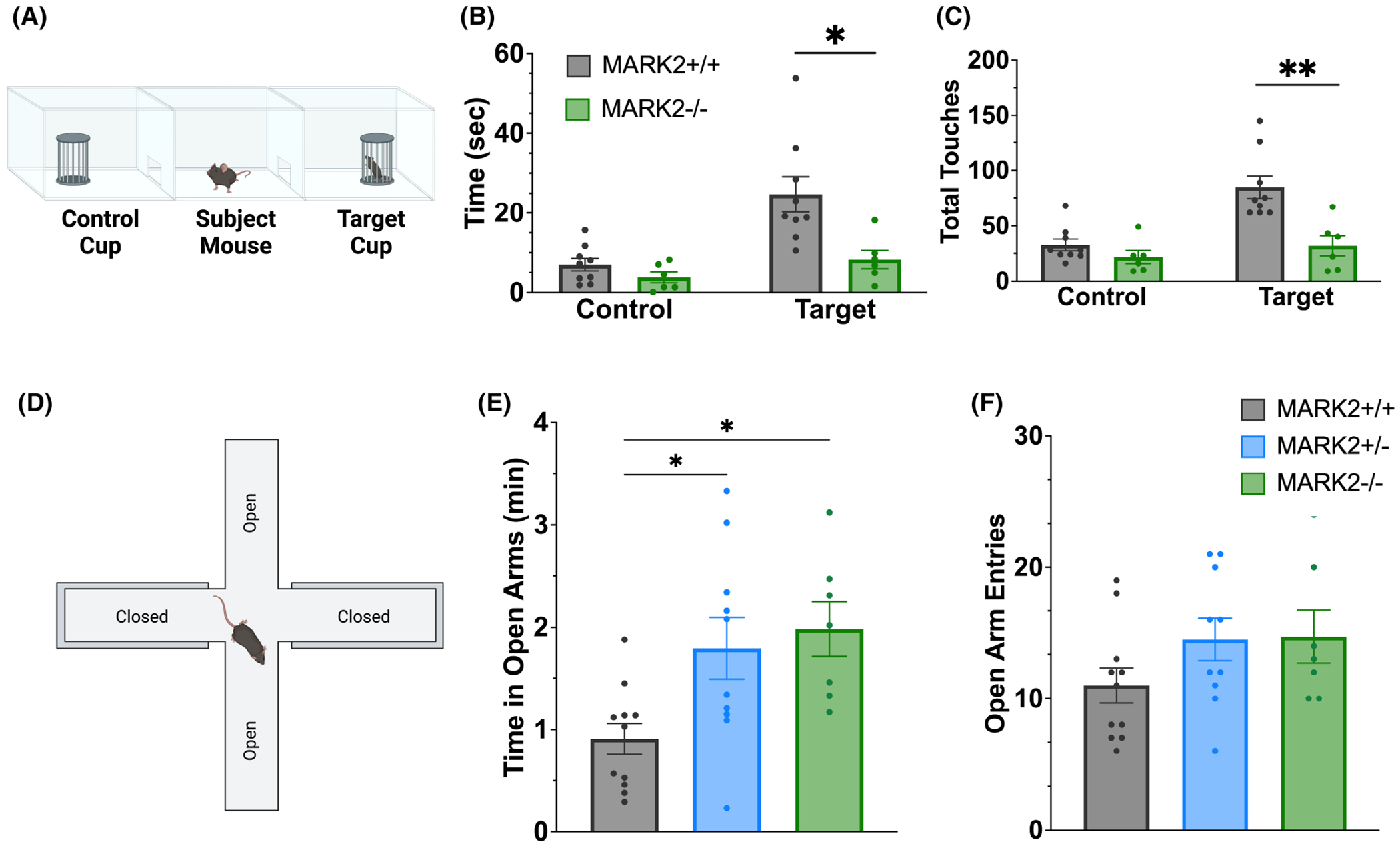
MARK2−/− mice show reduced sociability and reduced anxiety-like behavior. (A) Diagram of three-chamber sociability test. Mice were habituated to the chamber for 10 min. After habituation, an age- and sex-matched MARK2+/− mouse was placed in the target cup and the subject mouse was placed back into the center chamber. The control cup was left empty. (B) Total amount of time MARK2+/+ (*n* = 9) and MARK2−/− (*n* = 6) subject mice interacted with the control and target cups. Unpaired *t*-test was used to test for differences between MARK2+/+ vs. MARK2−/− for control (*t* = 1.451, *p* = .1704) and target cups (*t* = 2.822, *p* = .0144). (C) Total number of times MARK2+/+ and MARK2−/− subject mice touched the control and target cups with one or both paws. Mann–Whitney *U*-test was used to test for differences between MARK2+/+ vs. MARK2−/− for control (*U* = 11.50, *p* = .0692) and target cups (*U* = 3, *p* = .0028). (D) Diagram of the EPM. Each mouse was placed in the center square and observed for 10 min. (E) The total amount of time MARK2+/+ (*n* = 13), MARK2+/− (*n* = 12) and MARK2−/− (*n* = 8) spent in the open arms. One-way ANOVA (*F*(2, 25) = 5.743, *p* = .0088) followed by Dunnet’s *post-hoc* test. (F) The total number of open arm entries of MARK2+/+, MARK2+/−, MARK2−/− mice. An entry was defined as when all four paws entered the open arm. One-way ANOVA *F*(2, 25) = 1.796, *p* = .1868). *p < .0332, **p < .0021. Plot shows mean +/− SEM. Full statistics details can be found in Table S1–1.

Altered dendritic spine morphology and synaptic transmission have been shown to coincide with deficits in social interaction and altered anxiety levels.^57–59^ In addition, MARK2 is associated with neurological disorders that often exhibit social deficits and anxiety.^15–18,20,22,23,60^ Therefore, we wanted to explore whether MARK2−/− mice exhibit deficits in these behaviors using the three-chamber social interaction test. Subject mice were placed into the center chamber with an empty cylinder in one of the end chambers (control) and a cylinder containing a sex- and age-matched heterozygous mouse target in the other end chamber (target) (Figure 4A). To measure social interaction, the number of times the subject mouse contacted each cylinder and the total amount of time the subject mouse spent interacting with each cylinder were recorded. Intriguingly, we found that MARK2−/− mice exhibited a significant decrease in the amount of time spent interacting with the target compared to MARK2+/+ controls (Figure 4B, Table S1–1), and significantly fewer target cup touches compared to the MARK2+/+ mice (Figure 4C). Together, these results suggest that MARK2−/− mice exhibit impaired social interactions and that MARK2 plays an important role in regulating social behavior.

Next we examined whether anxiety was affected in MARK2−/− mice. To test the anxiety level of MARK2−/− mice, we used the EPM), which measures the anxiety induced by open spaces and height. Mice were placed in the center of the plus-shaped maze, which consisted of two long closed arms and two short open arms (Figure 4D). The number of times and duration of time spent in the open arms were recorded. Surprisingly, we found that MARK2+/− and MARK2−/− mice spent more time in the open arms compared to controls (Figure 4E). The number of open arm entries trended towards an increase in MARK2+/− and MARK2−/− mice, but did not reach significance (Figure 4F). The increased time spent in open arms with partial and total knockout of MARK2 suggests that MARK2−/− mice exhibit less anxiety.

### 3.5 | Heterozygous deletion of MARK2 results in increased seizure susceptibility and neuronal hyperexcitability

**FIGURE 5:**
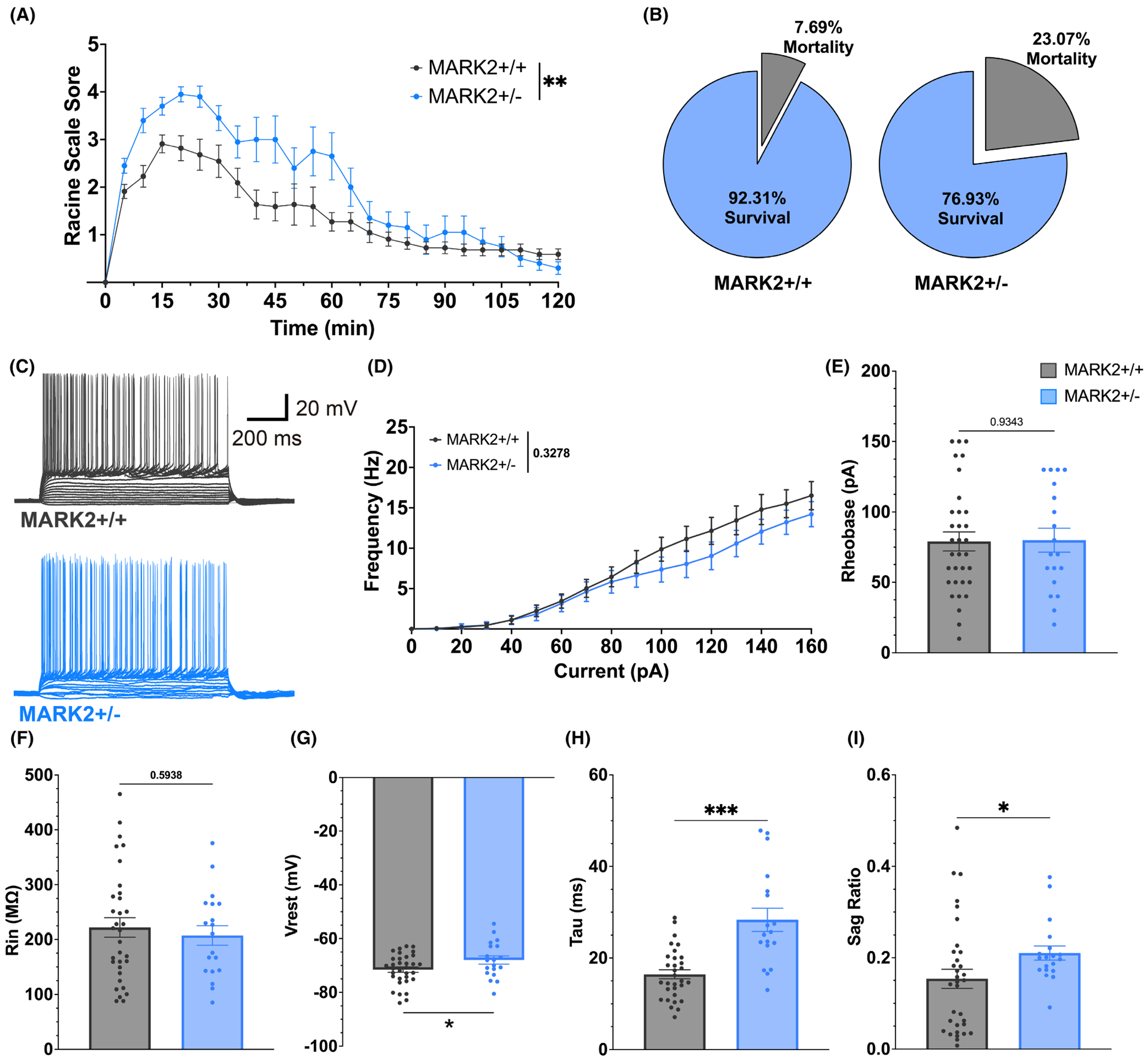
Heterozygous deletion of MARK2 results in increased seizure susceptibility and neuronal hyperexcitability. (A) MARK2+/+ (*n* = 13) and MARK2+/− (*n* = 14) mice were injected with pilocarpine (300 mg/kg) to induce seizures. Methyl scopolamine (1 mg/kg) was injected 30 min prior to pilocarpine to minimize peripheral cholinergic side effects. Mice were observed for 2 h and scored on the Racine scale by a blinded experimenter. Two-way repeated measures ANOVA (Genotype: *F*(1, 19) = 8.641, *p* = .0084). (B) Mortality rate among mice used for seizure susceptibility test. (C) Representative traces of action potentials from MARK2+/+ and MARK2+/− neurons in response to current injection. (D) Input–output curves showing the frequency of action potentials in response to increasing current injections in MARK2+/+ (*n* = 33) and MARK2+/− (*n* = 19) neurons. Two-way ANOVA (Genotype: *F*(1, 50) = 0.9767, *p* = .3278). (E) Comparison of rheobase (the minimum current required to elicit an action potential) between MARK2+/+ (*n* = 33) and MARK2+/− (*n* = 19) neurons. Unpaired *t*-test (*t* = 0.083, *p* = .9343). (F) Input resistance (Rin) shows no significant difference between MARK2+/+ (*n* = 33) and MARK2+/− (*n* = 19) neurons. Unpaired *t*-test (*t* = 0.537, *p* = .5938). (G) Resting membrane potential (Vrest) is significantly different between MARK2+/+ (*n* = 33) and MARK2+/− (*n* = 19) neurons. Unpaired *t*-test (*t* = 2.045, *p* = .0462). (H) Membrane time constant (Tau) is significantly different between MARK2+/+ (*n* = 33) and MARK2+/− (*n* = 19) neurons. Welch’s *t*-test (*t* = 4.386, *p* = .0002). (I) Sag ratio, indicative of membrane potential rectification, is significantly different between MARK2+/+ (*n* = 33) and MARK2+/− (*n* = 19) neurons. Mann–Whitney *U* test (*U* = 184, *p* = .0133). *p < .0332, **p < .0021, ***p < .0002. Mean +/− SEM. Full statistics details can be found in Table S1–1.

Seizures are often found to be comorbid with neurological disorders such as ASD,^3,4,61–64^ schizophrenia,^65,66^ and AD,^6–8,67,68^ all of which have been genetically linked to MARK2. Additionally, *de novo* MARK2 loss-of-function mutations have been linked to neurodevelopmental disorders with epilepsy.^21^ To determine whether the loss of MARK2 affects seizure susceptibility, MARK2+/+ and MARK2+/− mice were injected with pilocarpine (300 mg/kg) intraperitoneally to induce seizures. To reduce the side effects from peripheral cholinergic inputs, mice were also injected with methyl scopolamine (1 mg/kg) 30 min prior to pilocarpine injection. Mice were recorded for 2 h following the pilocarpine injection and seizure behavior was scored by a blinded observer using the Racine scale as modified by Borges et al.^31,32^ Unexpectedly, we found that the partial loss of MARK2 was enough to significantly increase seizure susceptibility (Figure 5A, Table S1–1). Furthermore, the mortality rate was three times higher in MARK2+/− mice compared to MARK2+/+ controls (Figure 5B). Since this phenotype was strong in MARK2+/− mice and the mortality rate was significantly elevated compared to MARK2+/+ mice, we did not perform this experiment on MARK2−/− mice as the result would have likely been much more fatal. Altogether, these results suggest that the partial loss of MARK2 is sufficient to result in increased seizure susceptibility.

This result is surprising given our findings that MARK2+/− and MARK2−/− mice have a shift towards immature dendritic spines and reduction in excitatory transmission. To further probe for changes in neuronal physiology that might explain the observed increase in seizure susceptibility, we characterized active and passive electrophysiological properties of dorsal hippocampal CA1 neurons of 5- to 7-week-old MARK2+/+ and MARK2+/− mice. Using whole-cell patch clamp recordings, we characterized the frequency-current (F-I) curves of action potentials and rheobase. Neither the F-I curve nor rheobase of MARK2+/+ and MARK2+/− mice showed a significant difference (Figure 5C–E).

Next, we characterized passive properties including the input resistance, resting membrane potential and membrane time constant. We found no significant change in the input resistance between MARK2+/+ and MARK2+/− mice (Figure 5F). Interestingly, the resting membrane potential showed significant depolarization in MARK2+/− mice (Figure 5G), in addition to an increased membrane time constant (Figure 5H). Previous studies show that hyperpolarization-activated cyclic nucleotide-gated (HCN) cation channels not only play a large role in setting the resting membrane potential,^69–71^ but enhanced HCN current has been shown to contribute to increased neuronal excitability in epilepsy.^72^ Therefore, we calculated the sag ratio as a measure of HCN channel current and found that the sag ratio was significantly increased in MARK2+/− mice (Figure 5I). Together, these results indicate that the loss of MARK2 results in increased intrinsic neuronal excitability, which manifests as seizures at the behavioral level.

### 3.6 | MARK2−/− mice exhibit transcriptional dysregulation of ion channels

**FIGURE 6:**
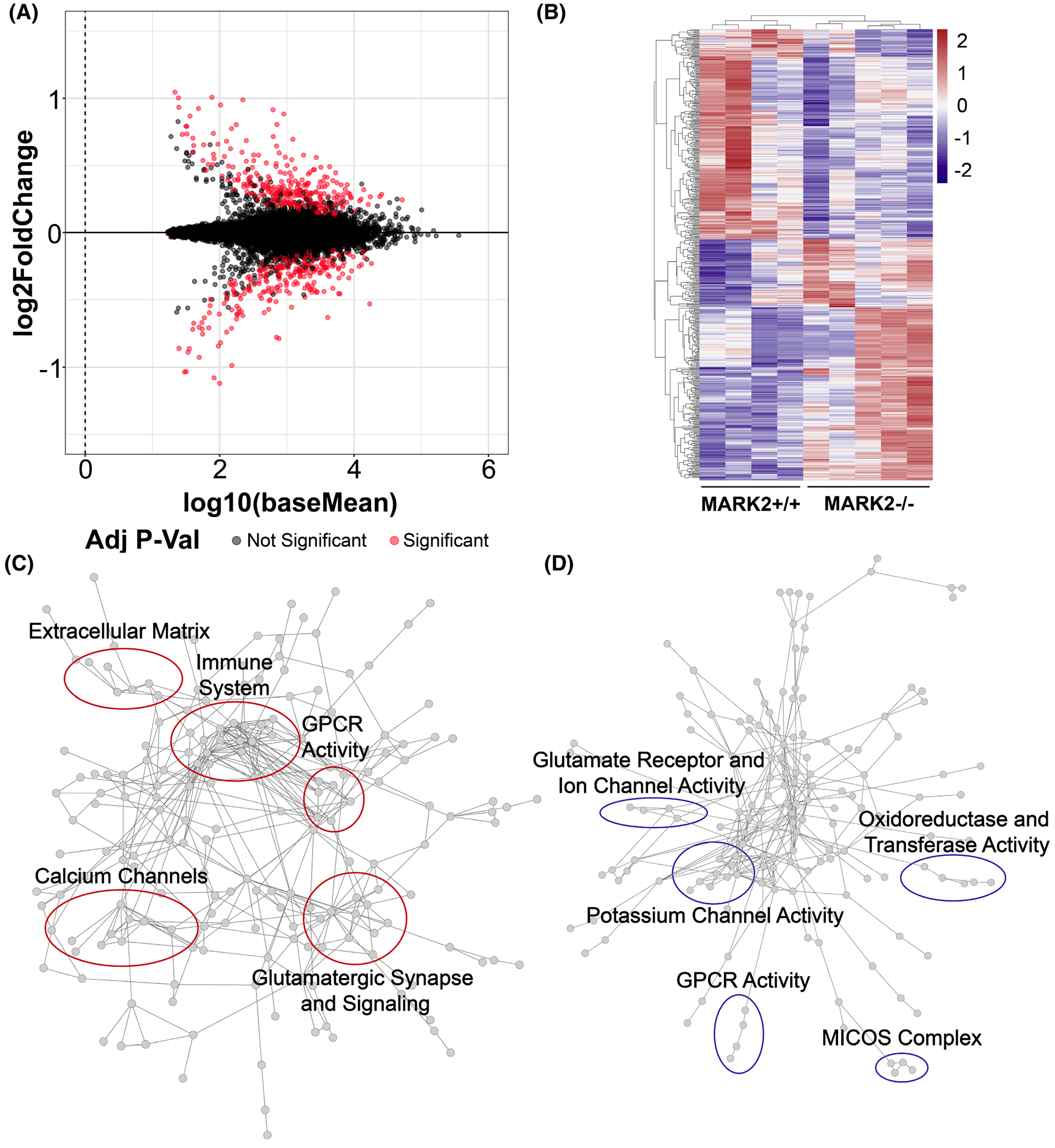
MARK2−/− mice exhibit the transcriptional dysregulation of ion channels. (A) MA plot of 15 625 genes from DESeq2 analysis of 8-week-old MARK2+/+ (*n* = 4) and MARK2−/− (*n* = 5) mouse hippocampi. Significant genes are shown in red (*p*_adj_ < .05). See Table S6–1 and S6–2 for full DESeq2 output. (B) Heatmap of all significant upregulated and downregulated genes identified in DESeq2 analysis. (C, D) Networks of upregulated (C) and downregulated (D) genes were made using the STRING database in Cytoscape. The largest subnetwork was selected for subsequent clustering analysis with MCL Cluster AutoAnnotate. The top 5 clusters are circled and labeled with the GO (molecular function) term most associated with the genes in each cluster.

To gain further insight into the molecular mechanisms that may contribute to the behavioral phenotypes observed with the loss of MARK2, we performed unbiased bulk RNAseq on MARK2+/+ and MARK2−/− hippocampi. We used DESeq2 to perform differential expression analysis and found 522 differentially expressed genes (Figure 6A,B, Table S6–1 and S6–2). Among these changes, we observed a significant increase in *Mark2* transcript levels in the MARK2−/− hippocampi; however, we confirmed that exons 2–4 were removed, which should prevent these transcripts from encoding a functional protein (Figure S4). We observed no significant changes in mRNA levels of *Mark1*, *Mark3* and *Mark4*, which is consistent with our Western blot analyses of MARK proteins (Figures 1 and S4). This further confirms that there are no compensatory gene expression changes in other MARK family members.

**FIGURE 7:**
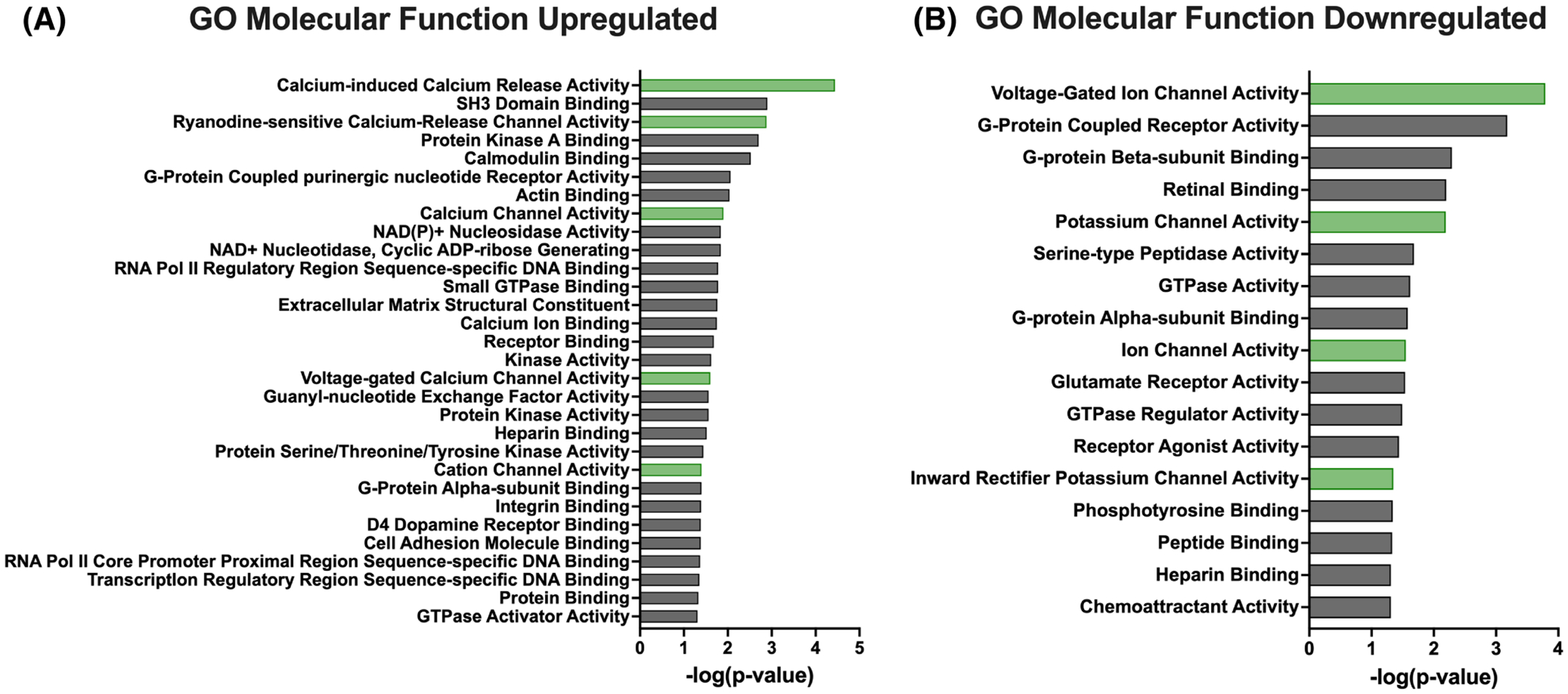
GO analysis in MARK2−/− mice. (A, B) GO (molecular function) analysis was conducted with DAVID. Terms are sorted in order significance according to the adjusted *p*-value. Green bars indicate terms associated with ion channels and ion homeostasis. See Table S7–1 for full GO output from DAVID.

To determine which molecular pathways are dysregulated in the MARK2−/− mice, we created separate STRING networks of upregulated and downregulated genes followed by a clustering analysis to find the top 5 clusters in each network. Interestingly, we found that potassium channels and calcium channels are enriched in the upregulated and downregulated genes lists, respectively (Figure 6C,D). Furthermore, we found that glutamatergic and GPCR activity are enriched in both upregulated and downregulated genes (Figure 6C,D). To further understand the molecular functions enriched in our gene sets independent of their protein–protein interactions, we performed GO analysis in DAVID. We found that ion channels, particularly calcium and potassium channels, glutamatergic signaling, and GPCR activity are among the most enriched terms (Figure 7A,B, Table S7–1). The presence of ion channels and excitatory transmission machinery in our STRING and GO analyses indicates that dysregulation in these pathways likely underlie the behavioral and cellular phenotypes associated with the loss of MARK2.

## 4 | DISCUSSION

In this study, we aimed to understand how MARK2 contributes to synaptic connectivity and cognition in vivo. Interestingly, we found that MARK2−/− mice show an increase in immature dendritic spines and a reduction in mature mushroom-shaped spines. The observed spine changes are in line with our previous findings in primary hippocampal neurons and other reports in which MARK2 knockdown in vitro results in fewer mature spines and increased filipodia.^9,27^ To understand the functional impact these changes may have on synaptic transmission in MARK2+/− and MARK2−/− mice, we also performed electrophysiological recordings and found a decrease in mEPSC frequency, but no changes in amplitude. These results are consistent with our finding of a reduction in the number of mature (i.e., mushroom-shaped) spines and unaltered synaptosomal levels of postsynaptic density components. In addition, the lack of change in mEPSC amplitude suggests that the increased thin spines in MARK2+/− and MARK2−/− mice are likely not functionally mature and may not have AMPA receptors to mediate synaptic responses. Moreover, these results are in line with another study in which a peptide inhibitor targeting all four MARK family members in vitro results in reduced mEPSC frequency, but not amplitude.^73^

Changes in dendritic spine morphogenesis and synaptic transmission are often accompanied by alterations in synaptic plasticity. Interestingly, our previous studies show that phosphorylation of PSD-95 on Ser561 is important for bidirectional dendritic spine structural plasticity in primary hippocampal neurons.^74^ It will be interesting to explore how the loss of MARK2 in vivo impacts various forms of synaptic plasticity. Considering that NMDA receptor activation can elicit either long-term potentiation (LTP) or long-term depression (LTD),^75^ and MARK kinases are activated downstream of NMDA receptors,^76^ it is possible that the loss of MARK2 affects both LTP and LTD in vivo. It will also be interesting to see whether MARK2 is required for different phases of LTP or other forms of plasticity, which will need to be examined using more acute perturbations of MARK2 activity.

Given that MARK2 is implicated in neurological disorders such as ASD,^15–17^ bipolar disorder,^18^ epilepsy,^21^ schizophrenia,^19^ major depressive disorders,^20^ and AD^18,22,23^ we performed comprehensive behavioral analyses focusing on phenotypes observed in these disorders, including learning and memory, social behavior, and anxiety-related behaviors. Interestingly, we observed profound changes in hippocampal-dependent learning and memory as well as social behaviors in the MARK2−/− mice, which is consistent with the performance of mice from another MARK2 knockout mouse line (EMK1-knockout by gene trapping).^77^ Curiously, MARK2−/− mice show increased exploration of the open arm in the EPM test, indicating decreased anxiety-like behaviors. This is the opposite of the phenotype observed in ASD subjects and animal models, which typically show increased anxiety-like behaviors.^78–81^ However, increased open arm exploration has been observed in several AD mouse models.^82^ Similarly, FMR1 knockout mice, which is a model for Fragile X syndrome and exhibits core features of autism, show increased open arm exploration in EPM in several studies.^83–86^ It will be important in the future to examine how MARK2 activity changes in different neurological disorders such as ASD and AD. Furthermore, conditional knockout mice with brain region specific deletion of MARK2 will provide more insights into the role of MARK2 in different brain areas in controlling various behaviors.

We also examined seizure susceptibility in the MARK2 mice, as seizures are often comorbid with ASD, schizophrenia, and AD. Remarkably, even heterozygotic deletion of MARK2 results in a significant increase in seizure susceptibility, indicating altered network excitability. To further understand the underlying physiological mechanisms contributing to increased seizure susceptibility in MARK2+/− mice, we characterized active and passive properties of CA1 hippocampal neurons. We found that there was no difference in the F-I curves nor was there a change in rheobase in MARK2+/− mice. Notably, we found that MARK2+/− mice showed an increased membrane time constant and a more depolarized resting membrane potential. Previous studies show that leak, inward-rectifying, and voltage-gated potassium channels are highly related to resting membrane potential,^69–71^ suggesting that dysregulation of hyperpolarizing channels may underlie the increased membrane potential in MARK2+/− mice. Therefore, we probed for changes to HCN channel-mediated membrane potential changes and found that MARK2+/− mice exhibit an enhanced sag ratio. These results are consistent with reports that an increase in the sag ratio underlies seizures in epileptic rats.^87,88^ Taken together, our seizure susceptibility and electrophysiological data indicate that the loss of MARK2 leads to neuronal hyperexcitability, a phenotype often observed in epilepsy, anxiety,^89^ ASD,^4^ and neurodegenerative disorders.^8,90–92^

Finally, to explore additional molecular pathways affected as a result of MARK2 knockout, we performed an unbiased RNAseq analysis. Consistent with our spine analysis and electrophysiology results, we found significant dysregulation in genes associated with synaptic transmission and ion homeostasis. In particular, calcium and potassium channel genes were found to have increased and decreased expression, respectively, in MARK2−/− mice. This suggests that a change in the intrinsic membrane excitability due to disrupted ion homeostasis may underlie the seizure susceptibility in the MARK2−/− mice. Interestingly, MARK2 has been shown to phosphorylate and inhibit the two-pore potassium channel, TRESK.^93^ However this interaction would be predicted to result in a hyperpolarized membrane potential upon the loss of MARK2, which is the opposite of what we observed. Thus, other downstream targets are likely at play, and further studies built upon our RNAseq analysis are warranted to examine the mechanisms by which MARK2 regulates ion homeostasis and neuronal excitability.

What might be the molecular mechanisms underlying the dysregulation of gene transcription observed in MARK2−/− mice? While MARK2 is not known to be a transcriptional regulator, it has several substrates including CRTC2, CBP, and class IIa histone deacetylases (HDACs), which are transcriptional regulators.^94–100^ Since phosphorylation of CRTC2 and class IIa HDACs results in their restriction to the cytoplasm, the loss of MARK2 would theoretically lead to increased nuclear accumulation of these molecules. Additionally, since MARK2 negatively regulates CBP, the loss of MARK2 can lead to increased CBP activity.^96^ It will be interesting to explore the roles of these transcriptional regulators in modulating synaptic transmission and ion homeostasis in the MARK2−/− mice.

One limitation of our RNAseq experiment is that it is bulk RNA sequencing performed on whole hippocampal tissue. While the enrichment of synaptic transmission and ion homeostasis genes suggest a neuronal mechanism, many of these genes are also expressed in glial cells. Thus it is possible that dysregulation of gene transcription in glial cells also contributes to some of the observed phenotypes. Indeed, our previous studies found that the loss of MARK2 in microglia primes microglia during development and increases their sensitivity to injury.^101^ It will be of great interest to further examine the cell type specific changes in gene regulation in the MARK2−/− mice.

In conclusion, our study elucidates the in vivo functions of MARK2 in regulating neuronal connectivity and behaviors related to neurological disorders such as ASD and AD. We found that the loss of MARK2 results in learning and memory deficits, which are likely due to a shift towards immature dendritic spines and a reduction in excitatory synaptic transmission. Additionally, the partial loss of MARK2 is sufficient to result in increased seizure susceptibility, which can be explained by an increase in neuronal excitability in the CA1 region of the hippocampus. Furthermore, RNAseq analyses show gene dysregulation in synaptic transmission and ion homeostasis, which supports the behavioral and cellular phenotypes observed in this study. Further investigation into the specific molecular pathways and downstream targets of MARK2 will enhance our understanding of the underlying mechanisms and facilitate the development of targeted therapeutic strategies for neurological disorders associated with MARK2 dysregulation.

## Supplementary Material

Figures S1–S4

Table S1

Table S2

Table S3

Table S4

Additional supporting information can be found online in the Supporting Information section at the end of this article.

## Data Availability

The data that support the findings of this study are available in the supplemental material of this article and are available upon request from the corresponding author.

## Abbreviations:

ace-tubulin: acetylated tubulin
AD: Alzheimer’s disease
ASD: autism spectrum disorder
CBP: CREB binding protein
CRTC2: CREB regulated transcription coactivator 2
EPM: elevated plus maze
F-I Curve: frequency-current curve
GluR1: glutamate receptor subunit 1
GluR2: glutamate receptor subunit 2
GKAP: guanylate kinase associated protein
HDAC: histone deacetylase
HOMER1: homer protein homolog 1
HCN: hyperpolarization-activated cyclic nucleotide-gated channel
LWR: length-to-width ratio
LTD: long-term depression
LTP: long-term potentiation
MARK1: microtubule affinity regulating kinase 1
MARK2: microtubule affinity regulating kinase 2
MARK3: microtubule affinity regulating kinase 3
MARK4: microtubule affinity regulating kinase 4
mEPSCs: miniature excitatory postsynaptic currents
MWM: Morris water maze
Par1b: partitioning defective 1b
PSD95: postsynaptic density protein 95
VGlut1: vesicular glutamate transporter 1
