# Supplementary material for: Neuronal connectivity, behavioral, and transcriptional alterations associated with the loss of MARK2
Source: FASEB J. Author manuscript; Available in PMC 2026 Jun 16. (PMC13271256; doi:10.1096/fj.202400454R)
Supplement: Figures S1–S4 [file NIHMS2167548-supplement-Figures_S1_S4.pdf]

Supplemental Information

Neuronal connectivity, behavioral, and transcriptional alterations associated with the loss of MARK2

Hanna O. Caiola, Qian Wu, Junlong Li, Xue-Feng Wang, Shaili Soni, Kevin Monahan, George C. Wagner, Zhiping P. Pang, Huaye Zhang*

Supplemental Figures and Legends

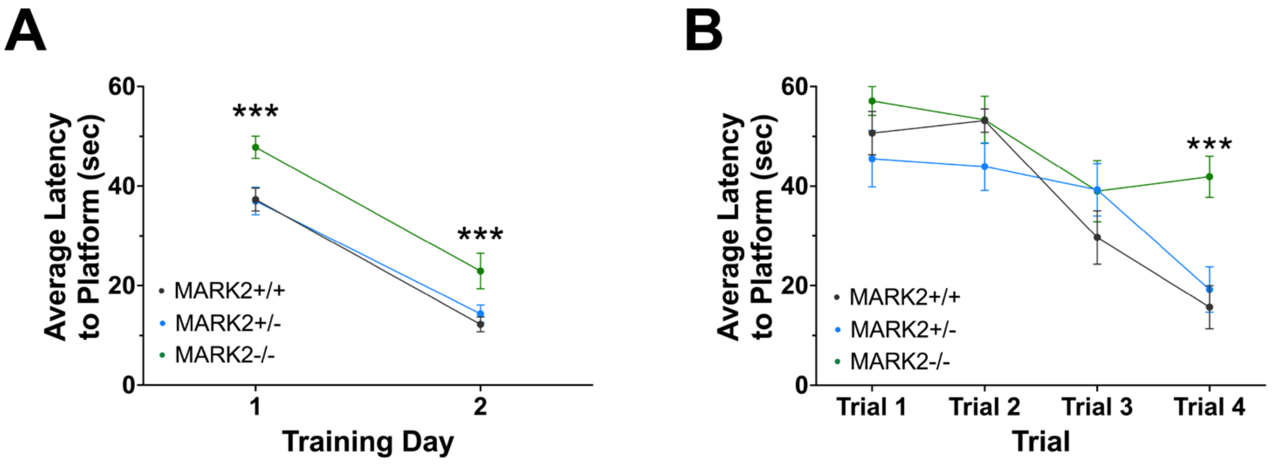

S1 Fig. Morris Water Maze Days 1 and 2

(A) Average latency to platform (in seconds) when platform was visible during training days 1 and 2. Two-way repeated measures ANOVA followed by Dunnett's *post hoc* test.

(B) Individual trials of training day 1. All mice successfully found the platform during the first three trials; however, MARK2-/- mice had increased latency by trial 4. Two-way repeated measures ANOVA followed by Dunnett's *post hoc* test.

*p<0.0332, **p<0.0021, ***P<0.0002, **** p<0.0001. Mean +/- SEM.

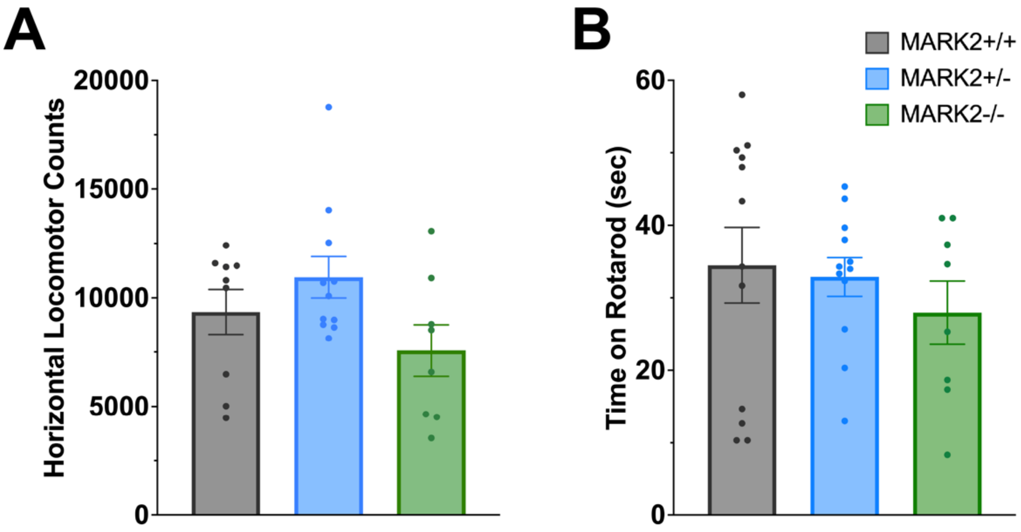

S2 Fig. Locomotor and Rotarod Tests

(A) No changes were observed in locomotor activity across genotypes. Activity was measured in MARK2+/+ (n=14), MARK2+/- (n=12) and MARK2-/- (n=10) mice by quantifying the number of times they crossed the photocell beams in the cage. Kruskall-Wallis (stat=3.171, p=0.2048).

(B) MARK2+/+ (n=14), MARK2+/- (n=12) and MARK2-/- (n=10) showed no differences in performance on the rotarod test. Brown Forsythe ANOVA (F(2, 26.1)=0.3080, p=0.7375). Mean +/- SEM plotted.

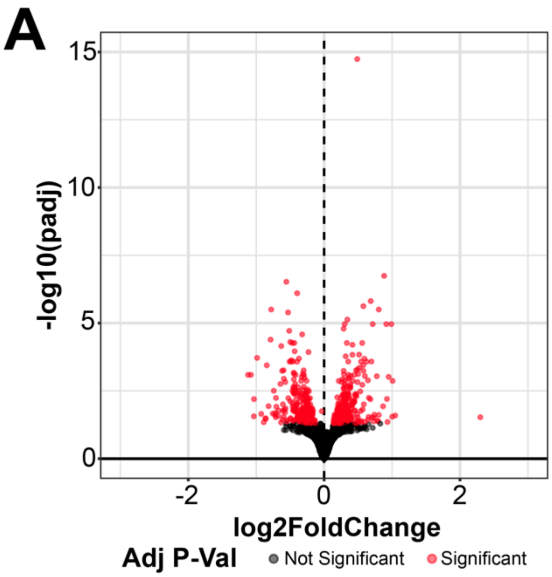

S3 Fig. Volcano Plot of bulk RNAseq of MARK2+/+ and MARK2-/- hippocampi.

(A) Volcano plot of 15,625 genes from DESeq2 analysis of 8-week-old MARK2+/+ (n=4) and MARK2-/- (n=5) mouse hippocampi. 522 significant genes are shown in red (Padj < 0.05).

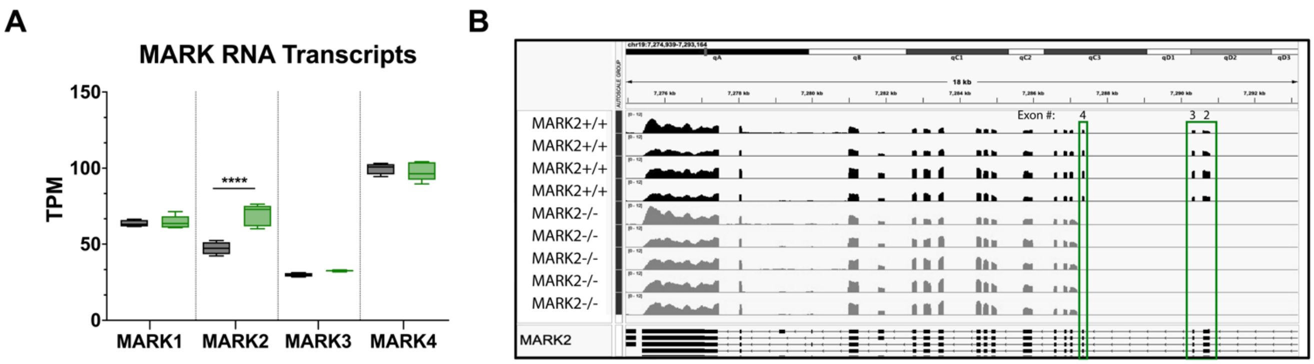

S4 Fig. MARK family gene expression from RNAseq.

(A) Transcripts per kilobase million (TPM) of MARK family genes in MARK2+/+ and MARK2-/- mice.

(B) Genome viewer showing *MARK2* gene in MARK2+/+ and MARK2-/- mice used for RNAseq. Green boxes indicate exons 2-4, which are absent from *MARK2* in the MARK2-/- mice.

S1 Table. Antibodies used in WB experiments

Antibody	Concentration	Catalog #	Vendor
MARK1	1:1000	21552-1-AP	ProteinTech
MARK2	1:1000	15492-1-AP	ProteinTech
MARK3	1:2000	05-680	Millipore
GAPDH (clone 6C5)	1:8000	MAB374	Sigma-Aldrich
GluA1 (clone N355/1)	1:1000	75-327	Neuromab
GluA2 (clone 6C4)	1:1000	MAB397	Sigma-Aldrich
PSD95 (clone K28/43)	1:2000	75-028	Antibodies, Inc. (NeuroMab)
SAPAP/GKAP	1:1000	73-156	NeuroMab
α-Tubulin	1:1000	AA4.3-S	DHSB
Acetylated Tubulin	1:8000	MABT868	Sigma-Aldrich
Homer1	1:1000	160 003	Synaptic Systems
VGlut1	1:2000	135 303	Synaptic Systems
Goat α-Rabbit HRP	1:5000	111-035-144	Jackson ImmunoResearch Laboratories
Goat α-Mouse HRP	1:5000	115-035-003	Jackson ImmunoResearch Laboratories

Extended Data

Table 1-1. Statistics Information. Excel file with information for each statistical test performed on all data described in the study.

Table 6-1. RNAseq DESeq2 Results. Excel file with full DESeq2 output for RNAseq experiment described in figure 6.

Table 6-2. RNAseq TPM Results. Excel file with TPM calculated for all genes identified in RNA sequencing experiment described in figure 6.

Table 7-1. DAVID Gene Ontology. Full gene ontology output (molecular function) for RNA sequencing experiment described in figures 6 and 7.
